# Haplotype analysis and marker development of five salt-tolerant–related genes in rice (*Oryza sativa* L.)

**DOI:** 10.3389/fpls.2023.1259462

**Published:** 2023-08-30

**Authors:** Pingbo Li, Zhen Li, Xu Liu, Hua Zhang, Shuyong Zhang, Fang Liu, Nana Li, Yongyi Yang, Kun Xie, Hanfeng Ding, Fangyin Yao

**Affiliations:** ^1^Institute of Wetland Agriculture and Ecology, Shandong Academy of Agricultural Sciences, Jinan, China; ^2^Agriculture and Rural Affairs Bureau of Yutai County, Jining, China; ^3^Institute of Crop Germplasm Resources, Shandong Academy of Agricultural Sciences, Jinan, China

**Keywords:** rice, salt tolerance, haplotype analysis, intragenic marker, germplasm accessions

## Abstract

Salinity stress is a great threat to the growth and productivity of crops, and development of salt-tolerant crops is of great necessity to ensure food security. Although a few genes with natural variations that confer salt tolerance at germination and seedling stage in rice have been cloned, effective intragenic markers for these genes are awaited to be developed, which hinder the use of these genes in genetic improvement of salt tolerance in rice. In this study, we first performed haplotype analysis of five rice salt-tolerant–related genes using 38 rice accessions with reference genome and 4,726 rice germplasm accessions with imputed genotypes and classified main haplotype groups and haplotypes. Subsequently, we identified unique variations for elite haplotypes reported in previous studies and developed 11 effective intragenic makers. Finally, we conducted genotyping of 533 of the 4,726 rice accessions from worldwide and 70 approved temperate *geng*/*japonica* cultivars in China using the developed markers. These results could provide effective donors and markers of salt-tolerant–related genes and thus could be of great use in genetic improvement of salt tolerance in rice.

## Introduction

Soil salinity is an important type of abiotic stress limiting the growth and productivity of crops worldwide. Rice, a staple food crop that feeds more than half of the world population, is susceptible to salinity stress during growth period, especially at germination stage and early seedling stage. Thus, development of salt-tolerant rice cultivars is of great necessity to the utilization of vast saline-alkaline land, which could further increase grain production and ensure food security (Yamaguchi and Blumwald, 2005; Qin et al., 2020). Salt tolerance in rice is a complex quantitative trait and regulated by many genes (Fan et al., 2021). Marker-assisted selection and pyramiding of salt-tolerant genes could be a promising way to improve salt tolerance of rice cultivars (Jiang et al., 2019; Shailani et al., 2021).

Up to now, large quantities of genetic studies on mining quantitative trait loci (QTL) and genes related to salt tolerance have been conducted, and hundreds of related QTL have been identified (Lin et al., 2004; Quan et al., 2018; Ju et al., 2022; Li et al., 2022; Lv et al., 2022; Yin et al., 2022; Yuan et al., 2022; Geng et al., 2023). However, only five genes with natural variations have been cloned (**Table 1**). *SKC1*/*OsHKT8*, the first cloned gene for salt tolerance at seedling stage, encodes a Na^+^ transporter (Ren et al., 2005). The allele of salt-tolerant accession ‘Nona Bokra’ carries four non-synonymous single-nucleotide polymorphisms (SNPs) relative to that of susceptible accession ‘Koshihikari,’ which affect transport properties of encoded proteins. *GS3*, a major gene for grain length and weight, encodes an atypical Gγ protein, which negatively regulates the phosphorylation of PIP2;1, leading to elevated reactive oxygen species (ROS) levels in rice under alkaline stress (Fan et al., 2006; Zhang et al., 2023). Thus, *GS3* is a negative regulator of salt tolerance at seedling stage. *RST1*/*OsARF18* encodes an auxin response factor (Deng et al., 2022). The protein encoded by elite haplotype *RST1^HapIII^* decreases repression activity of *OsAS1* and enhances salt tolerance at seedling stage. *OsHAK21* encodes a K^+^ transporter, and expression of the elite allele from salt-tolerant accession ‘Jiucaiqing’ was significantly induced by salinity stress in germinating seeds, which increased K^+^ and Na^+^ uptake, activated abscisic acid (ABA) signaling responses, and decreased the H_2_O_2_ level (He et al., 2019). *OsPAO3* encodes a polyamine oxidase, and expression of the elite allele from accession ‘Teqing’ was significantly induced by salinity stress in germinating seeds, which increased activity of polyamine oxidases, further eliminated over-accumulated H_2_O_2_, and finally resulted in stronger salt tolerance at the germination stage (Liu et al., 2022).

**Table 1:** Information of five salt-tolerant–related genes.

Gene symbol	LOC number	Protein encoding	Subcellular localization	Protein function	Salt tolerance
*SKC1/OsHKT8*	LOC_Os01g20160	A Na^+^ transporter belonging to the HKT family	Plasma membrane	Recirculate Na^+^ by unloading Na^+^ from the xylem and thus maintain a high K^+^/Na^+^ ratio in shoots	Seedling stage
*GS3*		An atypical Gγ protein	Plasma membrane	Block the interaction of another two Gγ proteins DEP1 and GGC2 with the Gβ protein RGB1 and thus repress signaling transduction	Seedling stage
*qSE3/OsHAK21*	LOC_Os03g37930	A K^+^ transporter belonging to the HAK family	Plasma membrane	Increase K^+^ uptake directly and Na^+^ uptake indirectly in germinating seeds	Germination stage
*qSTG4/OsPAO3*	LOC_Os04g53190	A polyamine oxidase		Increase polyamine content that enhances the activity of ROS-scavenging enzymes and promotes Na^+^ exclusion	Germination stage
*RST1/OsARF18*	LOC_Os06g47150	An auxin response factor	Nuclei	Repress the expression of gene *OsAS1*, leading to excess ammonium accumulation	Seedling stage

Development of effective markers for target genes is of great importance to marker-assisted selection (Zhang et al., 2020). However, effective intragenic markers for the five salt-tolerant–related genes are awaited to be developed. In this study, haplotype analysis of the five genes was performed, and unique variations to elite haplotypes of the five genes were identified. Intragenic markers for the five genes were developed and were further exploited to perform genotyping of different panels of rice germplasm accessions. These results would be of great use in marker assisted improvement of salt tolerance in rice.

## Materials and methods

### Rice accessions and cultivars

Three panels of rice materials were exploited in this study. The first panel consisted of 38 rice accessions with reference genome, including *geng*/*japonica* (GJ) accession Nipponbare (NIP) (International Rice Genome Sequencing Project, 2005), well-known accessions Tetep (Wang et al., 2019) and Taichung Native 1 (Panibe et al., 2021), *xian*/*indica* (XI) accessions Minghui63 and Zhenshan97 (Song et al., 2021), XI accessions Huazhan and Tianfeng (Zhang et al., 2022), and 31 genetically diverse rice accessions (Qin et al., 2021) (**Supplementary Table 1**). The second panel consisted of 4,726 rice accessions, including 533 accessions (Chen et al., 2014), 950 accessions (Huang et al., 2011), 3,024 accessions (Wang et al., 2018), and 219 accessions (unpublished) from the website RiceVarMap v2.0 (http://ricevarmap.ncpgr.cn/) (**Supplementary Table 2**). The third panel consisted of 70 temperate GJ cultivars approved in northern provinces of China, which were stored in our laboratory (**Supplementary Table 3**). The subpopulation information of accessions in the first panel and the second panel referred to the work Wang et al. (2018) and the website RiceVarMap v2.0, respectively.

### Haplotype analysis

Genomic sequences of five salt-tolerant–related genes from 38 rice accessions with reference genome were extracted using the software BioEdit (Hall, 1999) and software SeqBuilder from the Lasergene package (DNASTAR, Inc., Madison, USA), including 2-kb region upstream of the start codon termed as 5′ untranslated region (UTR), the open reading frame (ORF), and 1-kb region downstream of the stop codon termed as 3′UTR. Sequence alignment for each gene was conducted using the software MEGA 7 with personal correction (Kumar et al., 2016) (**Supplementary Data**). For simplicity, imputed variation information of all above identified variations on exons of each gene from 4,726 accessions was downloaded from the website RiceVarMap v2.0 using corresponding variation IDs (Zhao et al., 2015).

**Figure 1:**
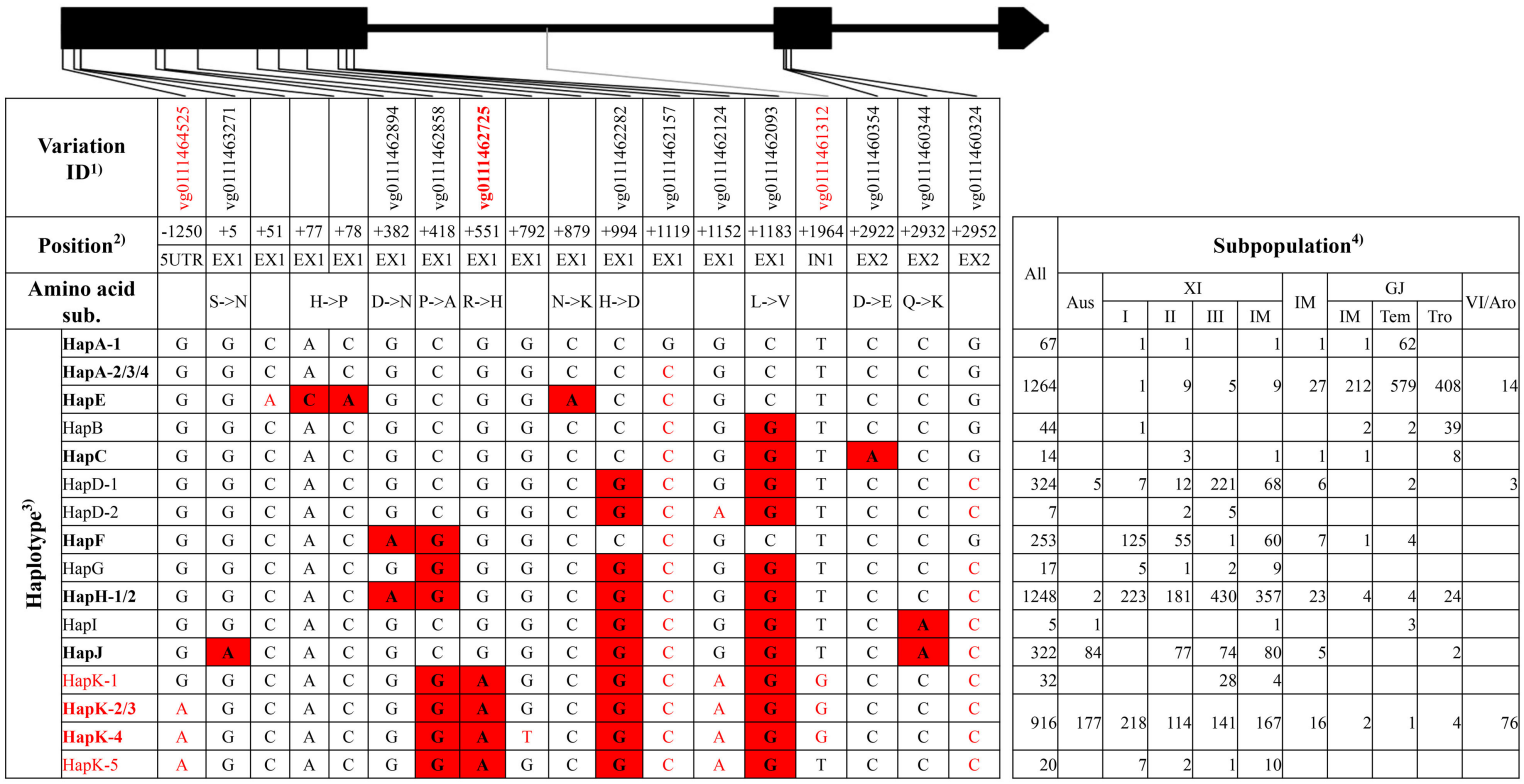
Haplotype analysis of gene *SKC1*/*OsHKT8.* Black boxes and lines represented exons and introns. 1) The variation IDs displayed were derived from the website RiceVarMap v2.0. The variation IDs in red color indicated unique variations to elite haplotype groups or haplotypes. 2) The position of each variation was the relative position to the start codon ATG, with A defined as “+1.” 5′UTR and EX represented 5′ untranslated region and exon, respectively. 3) The haplotypes or haplotype groups in bold were identified from the 38 rice accessions with reference genome, and that in red indicated elite haplotypes or haplotype groups. The black nucleotides filled with red color indicated non-synonymous SNPs to differentiate haplotype groups, and the red nucleotides indicated SNPs in untranslated regions or synonymous SNPs in coding region to differentiate different haplotypes in each group. 4) XI-IM, IM, GJ-IM, GJ-tem, GJ-tro and VI/Aro represented XI intermediate, Intermediate, GJ intermediate, temperate GJ, tropical GJ, and VI/Aromatic, respectively. The notes above were also suitable for the following four figures, namely, from **Figures 2**–**5**, and thus were not repeatedly displayed below.

**Figure 2:**
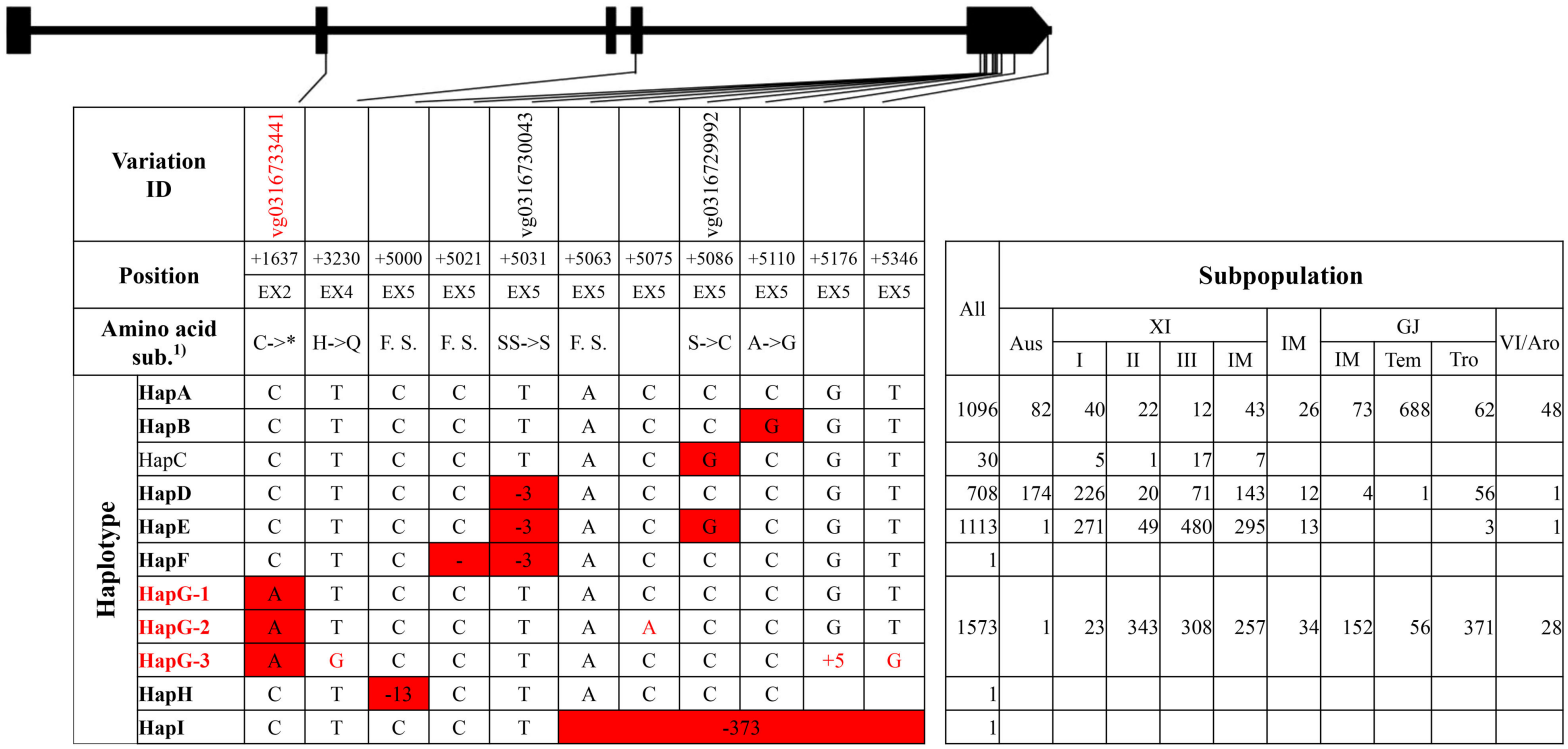
Haplotype analysis of gene *GS3.* 1) * indicated that the nucleotide A produced a stop codon. F.S. indicated that the 13-, 1-, and 373-bp deletion at position +5000, +5021, and +5063, respectively, caused frameshifts.

**Figure 3:**
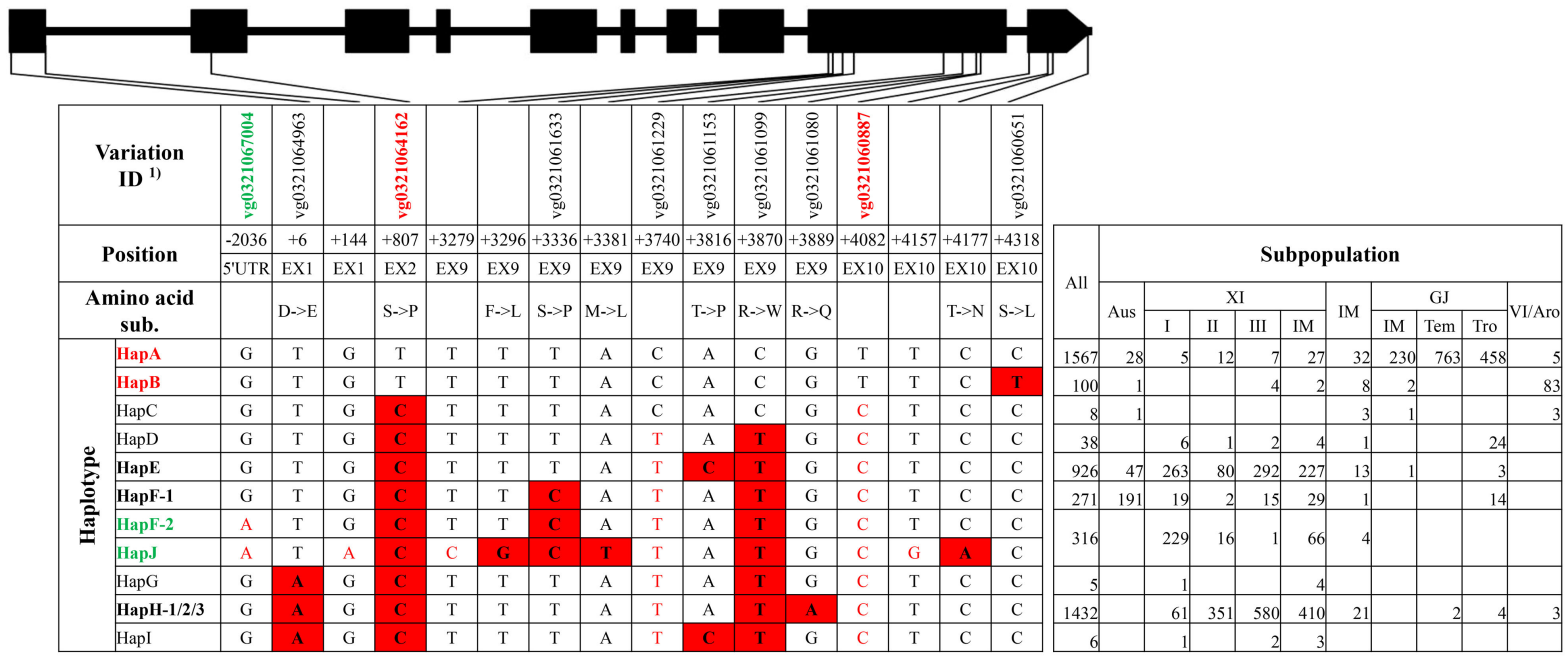
Haplotype analysis of gene *OsHAK21.* 1) The variation IDs in red color and green color indicated key variations to different elite haplotypes reported by He et al. (2019).

**Figure 4:**
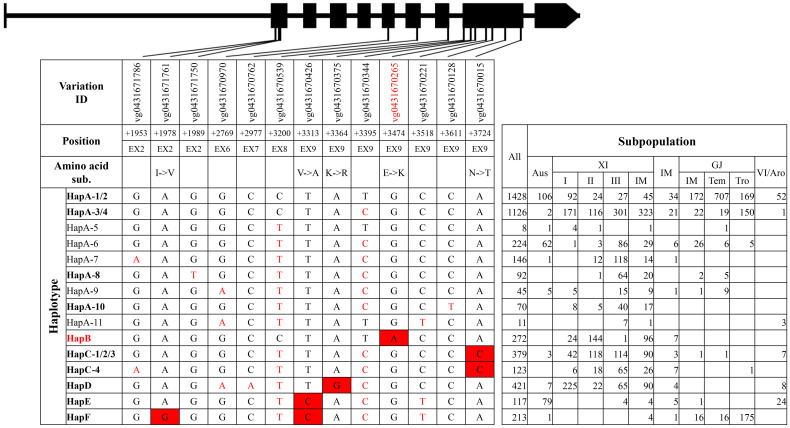
Haplotype analysis of gene *OsPAO3*.

**Figure 5:**
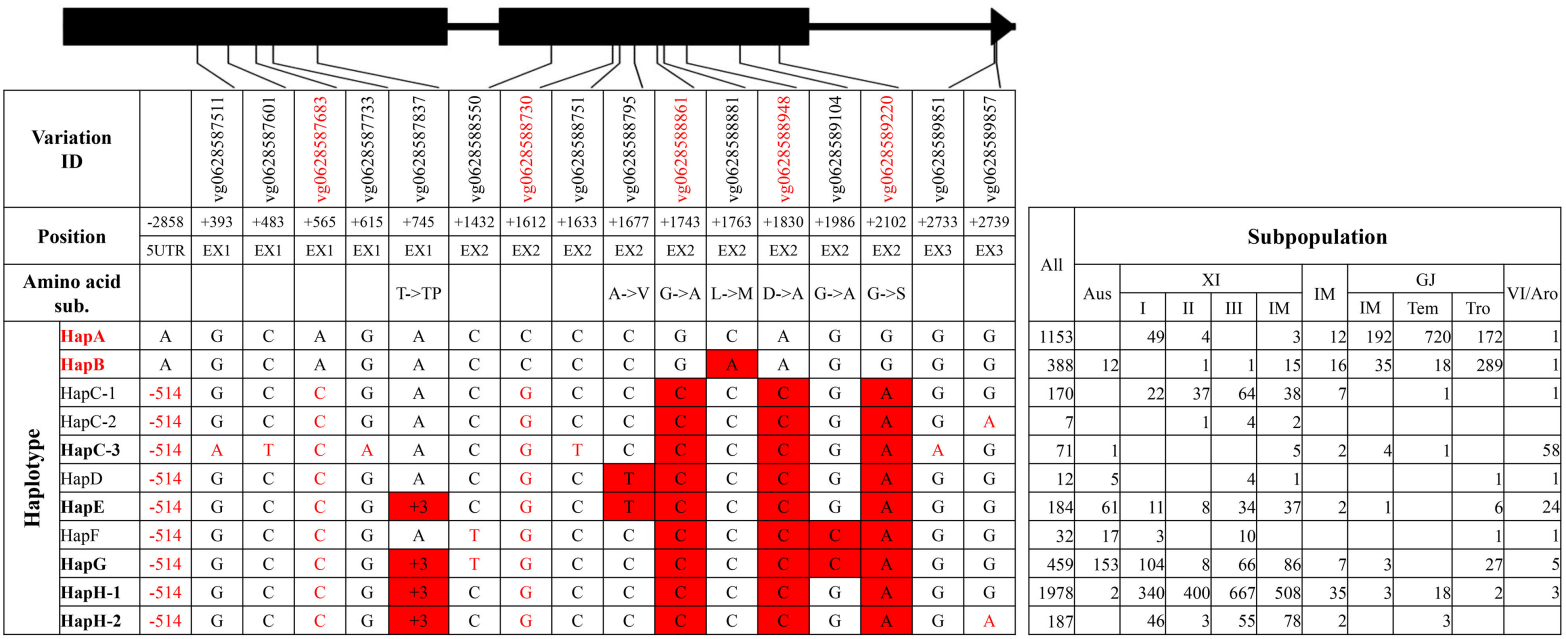
Haplotype analysis of gene *RST1*/*OsARF18*.

Haplotype analysis of each gene was conducted on the basis of a combination of all identified variations on genomic region of 38 rice accessions and imputed variations on exons of 4,726 rice accessions. For each gene, haplotype groups were classified on the basis of variations on exons that caused amino acid substitutions, insertions and deletions, or produced frameshifts and premature stop codons. In each haplotype group, haplotypes were further classified on the basis of synonymous SNPs on exons and all variations in 5′UTR, introns and 3′UTR. The haplotype of accession NIP was defined as HapA or HapA-1, belonging to the haplotype group HapA, and elite haplotypes of each gene referred to published papers (**Figures 1**–**5**, **Supplementary Tables 1, 2, 4–13**). In addition, novel haplotype groups or haplotypes that were identified from imputed variations of 4,726 rice accessions and consisted of less than five accessions were omitted.

### Marker development

Several rules were followed during marker development. First of all, target variations should be unique to elite haplotypes or haplotype groups of each gene. The unique variations of each elite haplotype were identified using 4,726 accessions. Second, an insertion/deletion (Indel) whose size was between 7 and 30 bp was given priority, and an Indel marker with amplification fragment between 100 and 300 bp was developed. Alternatively, if the size of an Indel was larger than 100 bp, then a marker with amplification fragment between 400 and 1,000 bp could be developed. Third, if no appropriate Indel could be selected, a SNP that could be transformed into a common and cheap restriction enzyme site together with neighboring nucleotides was taken into consideration, and, then, a cleaved amplified polymorphic sequences (CAPS) marker with amplification fragment between 300 and 800 bp or a derived CAPS (dCAPS) marker with amplification fragment between 100 and 300 bp was developed.

Indel markers were developed using the software Primer Premier 6 (PREMIER Biosoft, San Francisco, USA). CAPS markers and dCAPS markers were developed using the website dCAPS Finder 2.0 (Neff et al., 2002) and software Primer Premier 6.

### Marker analysis

For all developed markers, the PCR reaction was 3 μL of genomic DNA with a concentration of 20 ng/μL, 0.5 μL of each primer with a concentration of 10 μM/L, 6 μL of ddH_2_O, and 10 μL of 2*Taq Master Mix P111 purchased from Nanjing Vazyme Biotech Co., Ltd. The PCR profile was 3 min at 94°C for denaturation, followed by 32 cycles of 94°C for 15 s, 55°C for 15 s, and 72°C for 15 s/kb; then by 3 min at 72°C for extension; and finally by 1 s at 26°C. The PCR products of CAPS markers and dCAPS markers were digested with corresponding restriction enzymes purchased from Takara Biomedical Technology (Beijing) Co., Ltd., according to the recommended reaction and temperature.

For PCR products of Indel markers and digested PCR products of CAPS and dCAPS markers, those with size smaller than 300-bp were run on 6% polyacrylamide gels with a voltage of 160 V for 120 min, and bands were then revealed using a silver staining procedure: Gels were washed for 1 min with dH_2_O, followed by incubated for 10 min in staining solution (0.4 g of AgNO_3_, 400 ml of dH_2_O) with gentle shaking, then washed two times for 1 min with dH_2_O, then incubated for 4 min in developing solution (6 g of NaOH, 2 ml of CH_3_CHO, 400 ml of dH_2_O) with gentle shaking, and finally washed for 1 min with water. Those PCR products with size larger than 300 bp were run on 2% agarose gels containing GeneRed nuclelic acid dye with a voltage of 120V for 20 min, and bands were developed in UVP EC3 Imaging System (Analytik Jena AG, Jena, Germany).

## Results

### Haplotype analysis of five salt-tolerant–related genes

To identify sequence variations in the five salt-tolerant–related genes as possible (**Table 1**), we extracted genomic sequences from 38 rice accessions with reference genome, including 2-kb 5′UTR, the ORF, and 1-kb 3′UTR; performed sequence alignment; and identified variations on all regions. For variations identified on exons, we downloaded imputed genotypes from the 4,726 germplasm accessions using corresponding variation IDs. Subsequently, we conducted haplotype analysis of each gene, with the type of accession NIP termed as HapA or HapA-1, belonging to the haplotype group HapA (see details in Materials and Methods section).

For *SKC1*/*OsHKT8*, 11 haplotype groups were identified (**Figure 1**, **Supplementary Tables 4, 5**). Among those, HapE and HapK-4 were represented only by accession ‘KY131’ and accession ‘R498,’ respectively, and totally carried five SNPs lacking of variation IDs on website RiceVarMap v2.0. HapK and HapH were the two primary haplotype groups in XI subpopulations, and HapA was the primary group in GJ subpopulations. HapD, HapF, and HapJ were mainly existed in XI subpopulations. Of all haplotype groups, HapK carried all four reported non-synonymous SNPs at position +418, +551, +994, and +1183 from salt-tolerant variety ‘Nona Bokra’ and was thus termed as the elite haplotype group (Ren et al., 2005).

For *GS3*, nine haplotype groups were identified (**Figure 2**, **Supplementary Tables 6, 7**). Eight variations at position +3230, +5000, +5021, +5063, +5075, +5110, +5176, and +5346 were lack of corresponding variation IDs on website RiceVarMap v2.0, of which six were carried only by an accession, such as the 1-bp deletion at position +5021 by accession ‘Chuan 7,’ the 13-bp deletion at +5000 by accession ‘Zhimali,’ and the 373-bp deletion at position +5063 by accession ‘SYB5’ (Fan et al., 2006; Zhang et al., 2021). HapA and HapB were the two primary haplotype groups in temperate GJ subpopulation, and HapD and HapE were the two primary haplotype groups in XI subpopulations. HapG was another major group in XI subpopulations and the major group in tropical GJ subpopulation. Of all haplotype groups, HapG carried the reported functional SNP that produced a stop codon at position +1637 from elite XI variety ‘Minghui63’ and was thus termed as the elite haplotype group (Fan et al., 2006; Mao et al., 2010).

For *qSE3*/*OsHAK21*, 10 haplotype groups were identified (**Figure 3**, **Supplementary Tables 8, 9**). Among those, HapJ was represented only by XI accession ‘DG’ and carried six SNPs on exons lacking of corresponding variation IDs. HapA was the primary haplotype group in GJ subpopulations, whereas HapE and HapH were primary haplotype groups in XI subpopulations (**Figure 3**). HapB, HapF-1, and HapF-2 were mainly existed in VI/aromatic, *aus*, and XI subpopulations, respectively. HapC, HapG, and HapI were rare haplotypes represented by less than 10 of 4,726 accessions. Of all haplotype groups, HapA and HapB carried all six reported SNPs at position +6, +807, +3740, +3870, +3889, and +4082 from salt-tolerant landrace ‘Jiucaiqing’ and were thus termed as elite haplotype groups (He et al., 2019). HapF-2 and HapJ carried the reported SNP at position −2036 from HAP3 and were thus likely to be other elite haplotypes (He et al., 2019).

For *qSTG4*/*OsPAO3*, six haplotype groups were identified (**Figure 4**, **Supplementary Tables 10, 11**). Eleven haplotypes were identified in group HapA, among which HapA-1 and HapA-2 were primary haplotypes in GJ subpopulations, whereas HapA-3 and HapA-4 were primary haplotypes in XI subpopulations (**Figure 4**). HapB, HapC, and HapD were mainly existed in XI subpopulations, whereas HapF was mainly existed in GJ subpopulations. Of all haplotype groups, HapB carried all three reported SNPs at position +3395, +3474, and +3724 from elite XI variety ‘Teqing’ and was thus termed as the elite haplotype group (Liu et al., 2022).

For *RST1*/*OsARF18*, seven haplotype groups were identified (**Figure 5**, **Supplementary Tables 12, 13**). HapA and HapB were two primary haplotype groups in GJ subpopulations, and HapG and HapH were two primary haplotype groups in XI subpopulations. HapC-1 and HapC-3 were mainly existed in XI subpopulations and VI/*aromatic* subpopulation, respectively. Of all haplotype groups, HapA and HapB carried all four reported SNPs at position +1743, +1830, +1986, and +2102 from elite haplotype RST1^HapIII^ and were thus termed as elite haplotype groups (Deng et al., 2022).

### Development of markers for salt-tolerant–related genes

**Figure 6:**
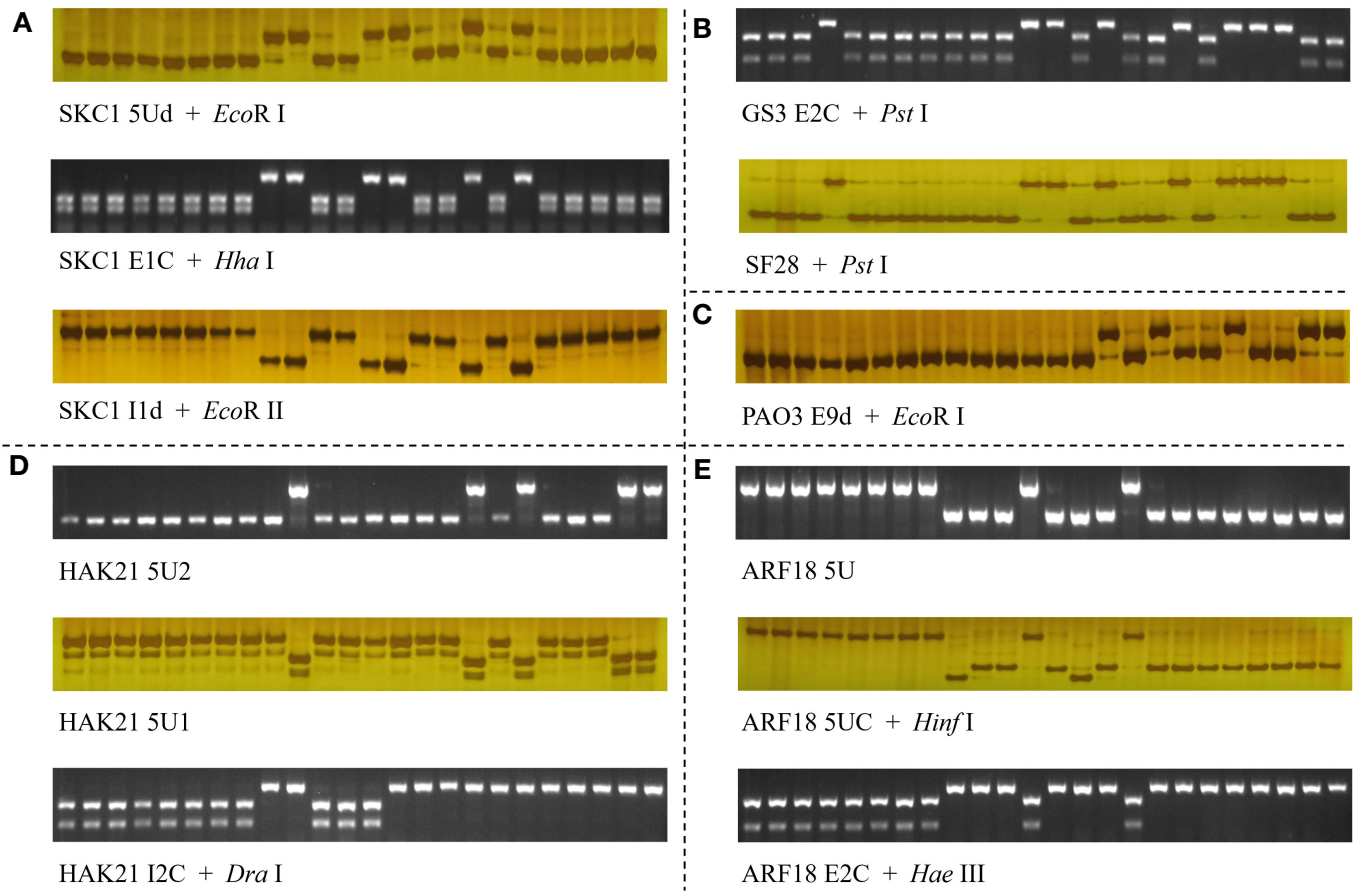
Molecular markers for five salt-tolerant–related genes, *SKC1*/*OsHKT8* **(A)**, *GS3* **(B)**, *OsPAO3* **(C)**, *OsHAK21* **(D)**, and *RST1*/*OsARF18* **(E)**. The 24 accessions used were NIP, Koshihikari, Kongyu131, Daohuaxiang No.2, Runnong 11, Shengdao 22, Nangeng 505, Zhonghua 11, Dular, Nanjing 11, 02428, Lemont, Basamati 370, Kasalath, Tetep, Suyunuo, Digu, Nona Bokra, Zhenshan 97, 9311, IR36, IR64, Huagengxian 74, and Guichao No.2. In **(B)**, the marker SF28 was developed by Fan et al. (2009).

To develop effective intragenic markers for salt-tolerant–related genes, we focused on the variations only carried by elite haplotype group of each gene; developed Indel, CAPS, and dCAPS markers (**Table 2**); and evaluated the effectiveness of these markers with 24 representative rice accessions (**Figure 6**).

**Table 2:** Molecular markers for the five salt-tolerant–related genes.

Gene	Variation position ^1)^	Variation ID ^2)^	Marker type	Marker name	Primer sequence (5′->3′)		Reference size ^3)^	Restriction enzyme	Haplotype ^4)^
*SKC1/OsHKT8*	−1250	vg0111464525	dCAPS	SKC1 5Ud	F	ACTGAGCATCTCAGATGATC	194/(20, 174)	*Eco*R I	HapK-2/3/4/5
					R	ACCGGCTAGAGTGGTGAATT			
	+551	vg0111462725	CAPS	SKC1 E1C	F	ACTCCTCCAAGATGATAGCA	423/(202, 221)	*Hha* I	HapK-1/2/3/4/5
					R	GAGACGACGGTGAAGATG			
	+1964	vg0111461312	dCAPS	SKC1 I1d	F	ACATGGATTGACGCTTcCAG	(20, 175)/195	*Eco*R II	HapK-1/2/3/4
					R	ATAACCTTCATCTAGCTAG			
*GS3*	+1637	vg0316733441	CAPS	GS3 E2C	F	AAAGTTGACAGGCTAAACAC	427/(130, 297)	*Pst* I	HapG-1/2/3
					R	GCTTGCACGATACTATGATT			
*qSE3/OsHAK21*	−2447		Indel	HAK21 5U2	F	TGACAGAGTGAGTGGCTAT	(+391)/262		HapF-2 & HapJ
					R	GCTGACTGGAATACTATGTAGA			
	−1169	vg0321066133	Indel	HAK21 5U1	F	AGGGTTTCACTTCTACTT	(-17)/179		HapF-2 & HapJ
					R	ACACTCGTACATCCACAAG			
	+1251	vg0321063718	CAPS	HAK21 I2C	F	GGTGACGGTTAGTGTTCTC	(172, 300)/472	*Dra* I	HapA & HapB
					R	TCTTCCGCCTGATGGTTA			
*qSTG4/OsPAO3*	+3474	vg0431670265	dCAPS	PAO3 E9d	F	CTCGACTCCAACTGAGAaTT	210/(20, 190)	*Eco*R I	HapB
					R	CCACCATTCCTTATTGCTAATC			
*RST1/OsARF18*	−2858		Indel	ARF18 5U	F	GGTGACGAGAGATCGGAC	866/(-514)		HapA & HapB
					R	CTGTATGCAAATTGGGAC			
	−1505	vg0628585614	CAPS	ARF18 5UC	F	TTATATGGCATTAGGCAATCC	195/(36, 159)	*Hinf* I	HapA & HapB
					R	TATACTCGCATGTGAAGGTT			
	+1612	vg0628588730	CAPS	ARF18 E2C	F	GGTTGGTCCTATGTGCTATT	(126, 315)/441	*Hae* III	HapA & HapB
					R	GTGCCATTCTGATTACTTCTC			

^1)^ Variation position is the position of target variation selected for marker development, relative to the start codon ATG.

^2)^ Variation ID is the ID of each target variation on the website RiceVarMap v2.0 (http://ricevarmap.ncpgr.cn/).

^3)^ The number before “/” is the size of fragment for elite haplotypes, whereas that following “/” is the size of fragment for other haplotypes. For Indel markers, the number in bracket is the information of target Indel carried by corresponding haplotypes. For CAPS and dCAPS markers, the number in bracket is the information of enzyme-digested products.

^4)^ Haplotypes represented elite haplotypes or haplotype groups detected with the developed marker.

For the elite group HapK of gene *SKC1*/*OsHKT8*, many unique variations were identified, including three SNPs and a 2-bp Indel in the 5′UTR, a SNP on exon 1, and five SNPs on introns (**Supplementary Tables 4, 5**). Among those, the SNP at position −1250 and neighboring nucleotides of non-elite haplotype groups could be transformed into a cut site for the restriction enzyme *Eco*R I, and, thus, a dCAPS marker ‘SKC1 5Ud’ was developed. The SNP at position +551 on exon 1 produced a cut site for the enzyme *Hha* I, and, thus, a CAPS marker ‘SKC1 E1C’ was developed. The SNP at position +1964 on intron 1 and neighboring nucleotides of elite haplotypes could be transformed into a cut site for the restriction enzyme *Eco*R II, and, thus, a dCAPS marker ‘SKC1 I1d’ was developed.

For the elite group HapG of gene *GS3*, the functional SNP at position +1637 was the only unique variation, which created a cut site for the restriction enzyme *Pst* I (**Supplementary Tables 6, 7**). A CAPS marker ‘SF28’ had been developed in a previous study (Fan et al., 2009). A CAPS marker ‘GS3 E2C’ for the same cut site was developed.

For the elite group HapA and HapB of gene *qSE3*/*OsHAK21*, 26 unique variations were identified, without consideration of the rare type HapC (**Supplementary Tables 8, 9**). These variations included six SNPs in the 5′UTR, three SNPs on exons, nine SNPs and three Indels on introns, and three SNPs in the 3′UTR. Among those, the SNP at position +1251 of elite groups created a cut site for the restriction enzyme *Dra* I, and, thus, a CAPS marker ‘HAK21 I2C’ was developed. For the elite haplotype HapF-2 and HapJ of gene *qSE3*/*OsHAK21*, seven unique variations were identified, including three Indels and two SNPs in the 5′UTR and a SNP on intron 1 (**Supplementary Tables 8, 9**). Two Indel markers ‘HAK21 5U2’ and ‘HAK21 5U1’ were developed for the 391-bp insertion and 17-bp deletion at position −2447 and −1169, respectively.

For the elite group HapB of gene *qSTG4*/*OsPAO3*, two unique variations were identified, which were the SNP in the 5′UTR at position −1141 and the SNP on exon 9 at position +3474 (**Supplementary Tables 10, 11**). Of the two, the SNP at position +3474 and neighboring nucleotides of non-elite haplotype groups could be transformed into a cut site for the restriction enzyme *Eco*R I, and, thus, a dCAPS marker ‘PAO E9d’ was developed.

For the elite group HapA and HapB of gene *RST1*/*OsARF18*, many unique variations were identified, including six SNPs and two Indels in the 5′UTR, five SNPs on exons, two SNPs on intron 2, and two SNPs in the 3′UTR (**Supplementary Tables 12, 13**). Among those, the 514-bp Indel at position −2858 was transformed into an Indel marker ‘ARF18 5U.’ The SNPs at position −1505 and +1612 created cut sites of *Hinf* I for non-elite haplotypes and *Hae* III for elite haplotypes, respectively, and, thus, two CAPS markers ‘ARF18 5UC’ and ‘ARF18 E2C’ were developed.

### Genotyping of salt-tolerant–related genes in germplasm accessions

To facilitate the development of salt-tolerant rice cultivars using related genes, we conducted genotyping of 533 rice accessions from the 4,726 accessions and 70 temperate GJ cultivars approved in China using the 11 markers developed above. The haplotypes of 533 rice accessions detected using markers were consistent with that using imputed SNPs, except for a few accessions (**Supplementary Table 14**). For the 70 approved temperate GJ cultivars, almost all cultivars carried elite haplotypes of *RST1*/*OsARF18* and *OsHAK21*, but not that of *SKC1*/*OsHKT8* and *OsPAO3* (**Supplementary Table 3**). In addition, only three of the 70 cultivars carried elite haplotype of *GS3*.

## Discussion

Development of effective markers for target genes is the premise of marker-assisted selection in genetic improvement. Intragenic markers are ideal markers for its co-segregating with elite alleles, whereas linked markers may lose its power due to possible crossover events between them and target genes. In this study, to ensure the effectiveness of developed markers, unique intragenic variations to elite haplotypes were identified using 4,726 rice germplasm accessions (**Supplementary Tables 3–12**), and, then, markers for appropriate variations were developed. Take the gene *SKC1*/*OsHKT8* for example. Five markers were developed in three granted patents in China and the corresponding variations were at position +418 (vg0111462858), +551 (vg0111462725), and +994 (vg0111462282), respectively (Zhou et al., 2018; Peng Y. B. et al., 2020; Peng P. et al., 2020). However, only the variation at position +551 (vg0111462725) was unique to elite haplotype group HapK. Three markers were developed in this study, and the marker ‘SKC1 E1C’ was designed for the variation at position +551. It was noteworthy that the variations of the other two markers were unique to some haplotypes of the elite group HapK. The 11 markers developed in this study could be of great use in marker-assisted genetic improvement of salt tolerance in rice breeding.

Asian cultivated rice could be classified into several subpopulations, and rice accessions in different subpopulations displayed a significant difference in agronomic traits, which was the result of pyramiding effects of different alleles or haplotypes (Huang et al., 2012). In this study, elite haplotypes of five salt-tolerant–related genes were distributed in different subpopulations (**Figures 1**–**5**). For the three genes conferring salt tolerance at seedling stage, elite haplotypes of *SKC1*/*OsHKT8* were mainly distributed in XI subpopulations, whereas that of *RST1*/*OsARF18* were mainly distributed in GJ subpopulations. The elite haplotypes of *GS3* were mainly distributed in XI and tropical GJ subpopulations. For the two genes conferring salt tolerance at germination stage, HapA of *OsHAK21* was mainly distributed in GJ subpopulations, whereas HapF-2 and HapJ were distributed in XI subpopulations. The elite haplotype of *OsPAO3* was mainly distributed in XI subpopulations. The dispersed distribution of elite haplotypes of the five genes in different subpopulations may provide an explanation for that the majority of rice accessions are susceptible to salt stress regardless of subpopulations. Thus, pyramiding of these elite haplotypes could be a promising way to improve salt tolerance of rice cultivars.

## Data availability statement

The original contributions presented in the study are included in the article/**Supplementary Material.** Further inquiries can be directed to the corresponding authors.

## Author contributions

PL: Writing – review & editing, Data curation, Formal Analysis, Funding acquisition, Investigation, Methodology, Resources, Software, Validation, Visualization, Writing – original draft. ZL: Writing – original draft, Formal Analysis, Investigation. XL: Writing – original draft, Formal Analysis, Investigation. HZ: Writing – original draft, Formal Analysis, Resources. SZ: Writing – original draft, Formal Analysis, Resources. FL: Writing – original draft, Formal Analysis, Resources. NL: Resources, Writing – original draft, Formal Analysis. YY: Formal Analysis, Funding acquisition, Resources, Writing – original draft. KX: Formal Analysis, Resources, Writing – original draft. HD: Conceptualization, Supervision, Writing – review & editing. FY: Conceptualization, Funding acquisition, Supervision, Writing – review & editing.
